# Crystallographic and spectroscopic characterization of 2-[(7-acetyl-4-cyano-6-hy­droxy-1,6-dimethyl-8-phenyl-5,6,7,8-tetra­hydro­isoquinolin-3-yl)sulfan­yl]-*N*-phenyl­acetamide

**DOI:** 10.1107/S2056989021000372

**Published:** 2021-01-15

**Authors:** Elham A. Al-Taifi, Islam S. Maraei, Etify A. Bakhite, Güneş Demirtas, Joel. T. Mague, Shaaban K. Mohamed, Youssef Ramli

**Affiliations:** a Department of Chemistry, Faculty of Science, Sana’a University, Sana’a, Yemen; b Department of Chemistry, Faculty of Science, Assiut University, Assiut, Egypt; c Ondokuz Mayıs University, Faculty of Arts and Sciences, Department of Physics, 55139, Samsun, Turkey; d Department of Chemistry, Tulane University, New Orleans, LA 70118, USA; e Chemistry and Environmental Division, Manchester Metropolitan University, Manchester M1 5GD, England; f Chemistry Department, Faculty of Science, Minia University, 61519 El-Minia, Egypt; g Laboratory of Medicinal Chemistry, Drug Sciences Research Center, Faculty of Medicine and Pharmacy, Mohammed V University in Rabat, Morocco

**Keywords:** crystal structure, tetra­hydro­iso­quinoline, phenyl­acetamide, hydrogen bond, C—H⋯π(ring) inter­action

## Abstract

In the title mol­ecule, C_28_H_27_N_3_O_3_S, the heterocyclic portion of the tetra­hydro­iso­quinoline unit is planar and an intra­molecular N—H⋯N hydrogen bond and a C—H⋯π(ring) inter­action help to determine the overall conformation. In the crystal, a layer structure with the layers parallel to (10) is generated by O—H⋯O and C—H⋯O hydrogen bonds.

## Abstract 2

The heterocyclic portion of the tetra­hydro­iso­quinoline unit is planar and an intra­molecular N—H⋯N hydrogen bond and a C—H⋯π(ring) inter­action help to determine the overall conformation. In the crystal, O—H⋯O hydrogen bonds form inversion dimers, which are connected by C—H⋯O hydrogen bonds, forming layers parallel to (10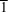).

## Chemical context   

Tetra­hydro­iso­quinolines exhibit important pharmacological activities including anti­tumor (Scott & Williams, 2002 ▸), anti­microbial (Bernan *et al.*, 1994 ▸), and dopamine­rgic activities (Andujar *et al.*, 2012 ▸). They are used as starting materials in the syntheses of pharmacologically active, constrained conformations of N-substituted-2-amino­pyridines as anti­nociceptive agents (Dukat *et al.*, 2004 ▸) and constrained conformations of nicotine to improve nicotine vaccines (Xu *et al.*, 2002 ▸; Meijler *et al.*, 2003 ▸; Carroll *et al.*, 2007 ▸). These examples demonstrate the utility of the tetra­hydro­iso­quinoline core and why these types of compounds are of great inter­est. In this context, we report here the synthesis and crystal structure of the title compound.
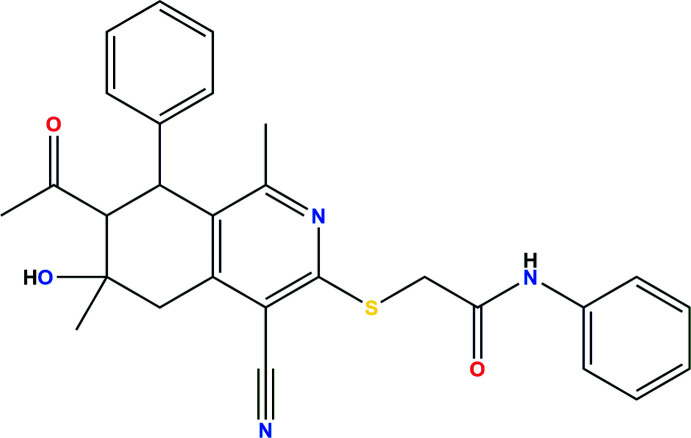


## Structural commentary   

**Figure 1:**
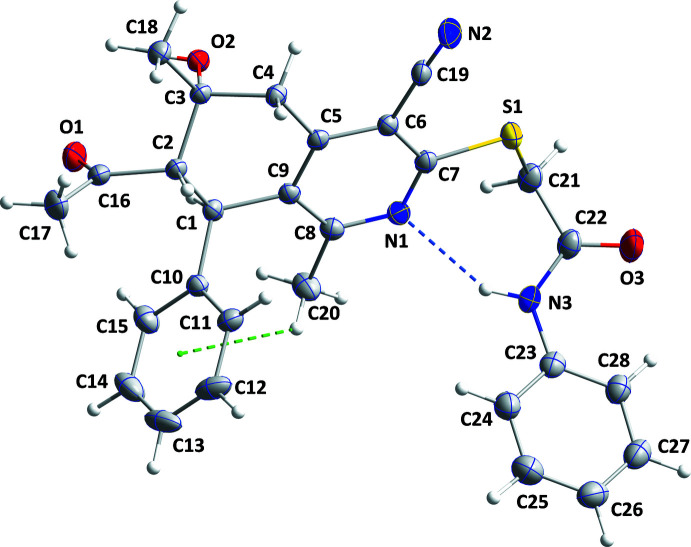
The title mol­ecule with labeling scheme and 50% probability ellipsoids. The intra­molecular hydrogen bond and C—H⋯π(ring) inter­action are depicted, respectively, by blue and green dashed lines.

The title compound crystallizes in space group *P*2_1_/*n* with one mol­ecule in the asymmetric unit (Fig. 1 ▸). The C5/C6/C7/N1/C8/C9 ring is approximately planar (r.m.s. deviation = 0.011 Å) with the largest deviation of 0.020 (1) Å being for atom C6. The best planes through the C10–C15 and C23–C28 rings are inclined to the above plane by 85.19 (6) and 64.22 (7)°, respectively. The orientation of the former ring is due in part to the C20—H20*A*⋯*Cg*3 (*Cg*3 is the centroid of the C10–C15 benzene ring) inter­action while the intra­molecular N3—H3*A*⋯N1 hydrogen bond affects the orientation of the second ring (Table 1 ▸ and Fig. 1 ▸) and places the two rings on the same side of the tetra­hydro­quinoxaline unit. The acetyl group on C2 is in an equatorial position while the hydroxyl group on C3 is axial and these are *syn* to one another. The C10–C15 ring attached to C1 is close to equatorial and *anti* with respect to both other subsituents (Fig. 1 ▸). Although the O2—H2*A* hydroxyl group is favorably oriented for forming an intra­molecular hydrogen bond with O1 as has been seen in some related mol­ecules (Mague & Mohamed, 2020 ▸), the H⋯O distance of *ca* 2.54 Å is long and a stronger, inter­molecular inter­action is favored (*vide infra*). A puckering analysis (Cremer & Pople, 1975 ▸) of the C1–C5/C9 ring yielded the following parameters: *Q* = 0.5267 (13) Å, θ = 128.52 (14)° and φ = 286.46 (18)°. The conformation of this ring approximates an envelope with C3 as the flap.

## Supra­molecular features   

**Figure 2:**
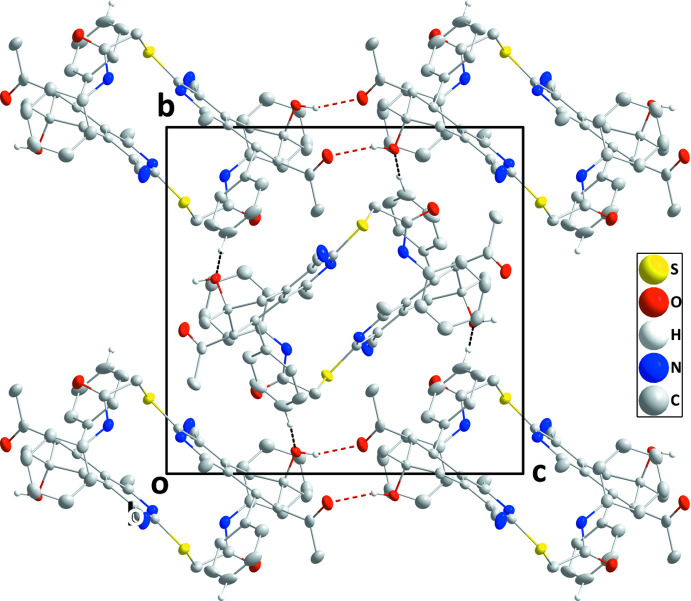
Packing viewed along the *a*-axis direction with inter­molecular O—H⋯O and C—H⋯O hydrogen bonds depicted, respectively, by red and black dashed lines.

In the crystal, inversion dimers are formed by O2—H2*A*⋯O1 hydrogen bonds (Table 1 ▸), which results in O1⋯O1^i^ and O1⋯O2^i^ [symmetry code: (i) −*x* + 1, −*y* + 1, −*z*] contacts of 2.8774 (16) and 2.8674 (14) Å (0.16 and 0.17 Å less than the sum of the van der Waals radii), respectively. The dimers are connected by C13—H13⋯O2 hydrogen bonds (Table 1 ▸), forming layers parallel to (10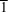) (Figs. 2 ▸ and 3 ▸).

## Database survey   

A search of the Cambridge Structural Database (CSD, updated to December 2020, Groom *et al.*, 2016 ▸) found three analogs of the title mol­ecule, one with a methyl group on sulfur (refcode AXUXOH; Dyachenko *et al.*, 2010 ▸) and two with a 4-chloro­phenyl group on C1 in place of the phenyl group, one with an ethyl group on sulfur (NAQRIJ; Mague *et al.*, 2017*a* ▸) and the other with a CH_2_CO_2_CH_3_ group on sulfur (PAWCEY; Mague *et al.*, 2017*b* ▸). In all three, the acetyl group is equatorial and the hydroxyl group is axial while the phenyl or 4-chloro­phenyl group is close to equatorial, as is the case with the title mol­ecule. The puckering amplitudes of the cyclo­hexene ring in the second and third mol­ecule are, respectively, 0.521 (2) and 0.524 (3) Å, which are essentially the same as in the title mol­ecule. One notable difference between the four mol­ecules is the orientation of the hydroxyl hydrogen. In AXUXOH there is an intra­molecular hydrogen bond with the acetyl group leading to an H⋯O distance of 2.23 Å. In the other three, inter­molecular hydrogen bonding of the hydroxyl group predominates and the intra­molecular H⋯O distances are 2.55, 2.71 and 3.18 Å for the title mol­ecule, PAWCEY and NAQRIJ, respectively.

## Hirshfeld surface analysis   

**Figure 4:**
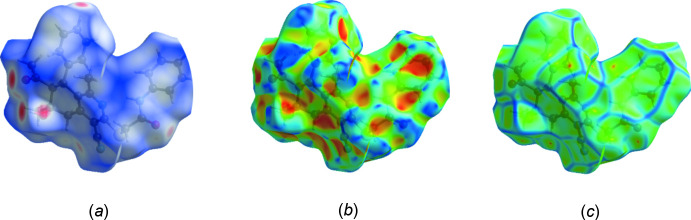
The Hirshfeld surface of the title mol­ecule mapped over (*a*) *d*_norm_, (*b*) shape-index, and (*c*) curvedness.

**Figure 5:**
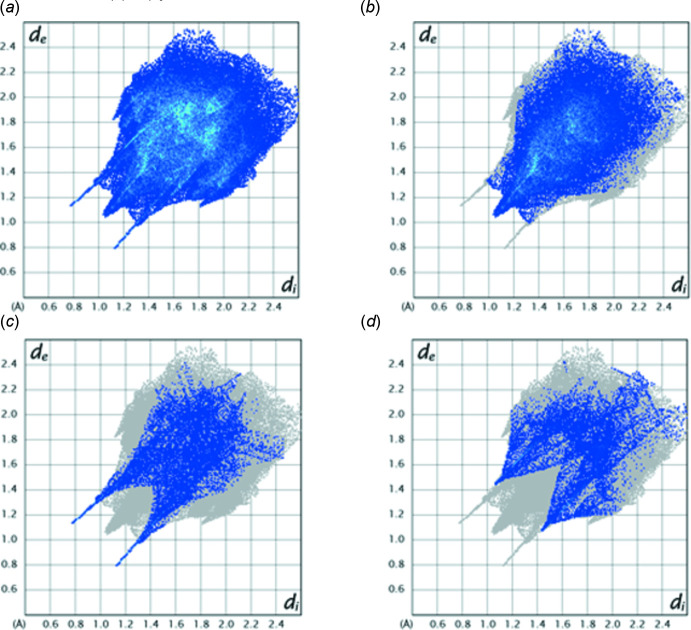
Fingerprint plots for the title mol­ecule showing (*a*) all contacts and those delineated into (*b*) H⋯H contacts, (*c*) H⋯O/O⋯H contacts, and (*d*) H⋯N/N⋯H contacts.

Hirshfeld surface analysis is an effective means of probing inter­molecular inter­actions (McKinnon *et al.*, 2007 ▸; Spackman & Jayatilaka, 2009 ▸), which can be conveniently carried out with *Crystal Explorer 17* (Turner *et al.*, 2017 ▸). A detailed description of the use of *Crystal Explorer 17* and the plots obtained is given by Tan *et al.* (2019 ▸). From the surface mapped over *d*_norm_ (Fig. 4 ▸ *a*), the sites of the inter­molecular O—H⋯O and C—H⋯O hydrogen bonds can be seen on the left side near the bottom and at the top, respectively. A weaker point of inter­action is at O3 on the lower right of the diagram, which might indicate a weak, inter­molecular C4—H4*B*⋯O3 hydrogen bond since the O⋯H distance is 2.605 (15) Å. The surfaces mapped over shape-index (Fig. 4 ▸ *b*) and curvedness (Fig. 5 ▸ *c*) show a relatively flat region over the C23–C28 benzene ring in the latter and a red triangular area over the edge of the ring in the former. This is suggestive of a C—H⋯π(ring) inter­action and can be identified with the C20—H20*A*⋯*Cg*3 inter­action noted in Section 2. The fingerprint plots derived from the Hirshfeld surface enable the apportionment of the inter­molecular inter­actions into specific sets. Fig. 5 ▸ *a* displays the plot for all inter­actions while Fig. 5 ▸ *b*–5*d* show those delineated into H⋯H, H⋯O/O⋯H and H⋯N/N⋯H inter­actions, which constitute 47.3%, 11.8% and 10.6% of the total inter­actions, respectively.

## Synthesis and crystallization   

A mixture of 7-acetyl-4-cyano-1,5-dimethyl-6-hy­droxy-8-phenyl-5,6,7,8-tetra­hydro­iso­quinoline-3(2*H*)-thione (10 mmol), *N*-phenyl-2-chloro­acetamide (10 mmol) and sodium acetate trihydrate (1.50 g, 11 mmol) in ethanol (100 mL) was heated under reflux for one hour. The precipitate that formed after standing at room temperature overnight was collected, washed with water, dried in air and then recrystallized from ethanol to afford the title compound in the form of colorless crystals. Yield: 4.00 g, 82%; m. p.: 470-472 K.

## Spectroscopic characterization   

The chemical structure of the compound has also been confirmed using analytical and spectroscopic methods. The FT–IR spectrum shows mainly the characteristic NH peak of the acetamide group at 3277 cm^−1^ and the C=O bond of the amide group at 1667 cm^−1^. In addition, characteristic peaks of the precursor are observed: OH at 3522 cm^−1^, aromatic C—H at 3058 cm^−1^, aliphatic C—H at 2920, 2970, 2991 cm^−1^, nitrile at 2217 cm^−1^ and acetyl at 1694 cm^−1^, also confirming the structure of the compound.

With regard to the ^1^H NMR spectrum, several characteristic signals can be clearly attributed to the title compound, such as a doublet of doublets between 4.09 and 4.19 ppm with a coupling constant of 16 Hz due to SCH_2 _, and a singlet at 10.22 ppm due to NH. In addition, we note the presence of characteristic peaks related to the starting compound: multiplets between 7.17 and 7.29 ppm due to aromatic protons, singlets at 1.28, 1.92, 2.11 and 4.84 ppm referring to a methyl group attached to a pyridine ring, the CH_3_ of the acetamide group and the hy­droxy group, respectively. The doublets between 7.53–7.55 (*J* = 8 Hz) and 7.02–7.04 (*J* = 8 Hz) can be attributed to the aromatic protons.

IR (cm^−1^): 3522 (OH); 3277 (NH); 3058 (C—H, aromatic); 2920, 2970, 2991 (C—H aliphatic); 2217 (C≡N); 1694 (C=O, acet­yl); 1667 (C=O, amide).

^1^H NMR (400 MHz, CDCl_3_): 10.22 (*s*, 1H, NH); 7.53–7.55 (*d*, *J* = 8 Hz, 2H, Ar-H); 7.23–7.29 (*m*, 4H, Ar-H); 7.17–7.20 (*m*, 1H, Ar-H); 7.02–7.04 (*d*, *J* = 8 Hz, 3H, Ar-H); 4.84 (*s*, 1H, OH); 4.52–4.54 (*d*, *J* = 8 Hz, 1H, CH at C-8); 4.09–4.19 (*dd*, *J* = 16 Hz, 2H, SCH_2_); 3.25–3.29 (*d*, *J* = 16 Hz, 1H, CH_2_); 2.94–2.96 (*d*, *J* = 8 Hz, 1H, CH at C-7); 2.89–2.94 (*d*, *J* = 20 Hz, 1H, CH_2_), 2.11 (*s*, 3H, COCH_3_); 1.92 (*s*, 3H, CH_3_ attached to pyridine ring); 1.28 (*s*, 3H, CH_3_).

## Refinement   

Crystal data, data collection and structure refinement details are summarized in Table 2 ▸. All hydrogen atoms were independently refined. Twelve reflections were not accessible due to the configuration of the goniometer and the low-temperature attachment.

## Supplementary Material

Crystal structure: contains datablock(s) global, I. DOI: 10.1107/S2056989021000372/vm2243sup1.cif

Structure factors: contains datablock(s) I. DOI: 10.1107/S2056989021000372/vm2243Isup2.hkl

Supporting information file. DOI: 10.1107/S2056989021000372/vm2243Isup3.cml

CCDC reference: 2055318

Additional supporting information:  crystallographic information; 3D view; checkCIF report

## Figures and Tables

**Figure 3:**
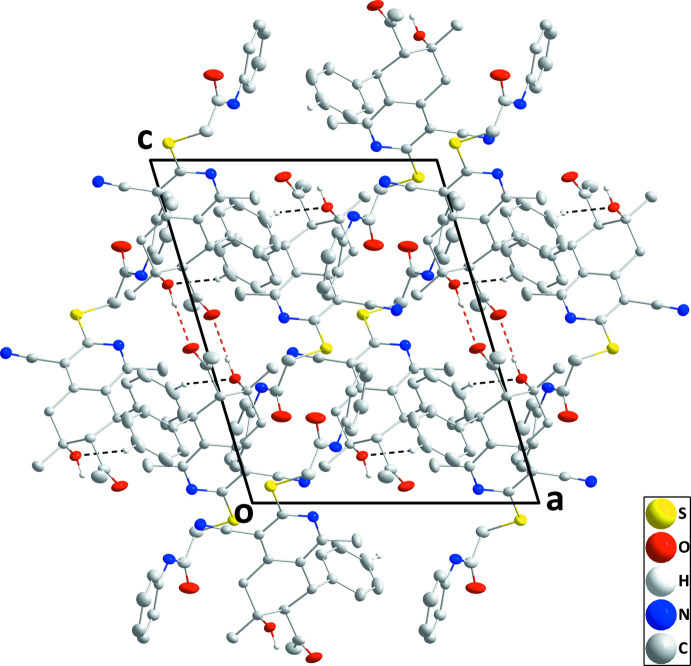
Packing viewed along the *b*-axis direction with inter­molecular O—H⋯O and C—H⋯O hydrogen bonds depicted, respectively, by red and black dashed lines.

**Table 1:** Hydrogen-bond geometry (Å, °) *Cg*3 is the centroid of the C10–C15 benzene ring.

*D*—H⋯*A*	*D*—H	H⋯*A*	*D*⋯*A*	*D*—H⋯*A*
O2—H2*A*⋯O1^i^	0.86 (2)	2.04 (2)	2.8674 (13)	161.9 (19)
N3—H3*A*⋯N1	0.887 (18)	2.306 (18)	3.1148 (15)	151.6 (15)
C13—H13⋯O2^ii^	0.94 (2)	2.40 (2)	3.2520 (19)	150.5 (17)
C20—H20*A*⋯*Cg*3	1.00 (2)	2.975 (19)	3.6866 (16)	128.7 (13)

Symmetry codes: (i) 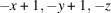; (ii) 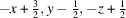.

**Table 2:** Experimental details

Crystal data	
Chemical formula	C_28_H_27_N_3_O_3_S
*M*_r_	485.58
Crystal system, space group	Monoclinic, *P*2_1_/*n*
Temperature (K)	150
*a*, *b*, *c* (Å)	12.0487 (4), 13.9821 (5), 15.0239 (5)
β (°)	106.606 (1)
*V* (Å^3^)	2425.46 (14)
*Z*	4
Radiation type	Cu *K*α
μ (mm^−1^)	1.47
Crystal size (mm)	0.26 × 0.14 × 0.08

Data collection	
Diffractometer	Bruker D8 VENTURE PHOTON 100 CMOS
Absorption correction	Multi-scan (*SADABS*; Krause *et al.*, 2015 ▸)
*T*_min_, *T*_max_	0.79, 0.89
No. of measured, independent and observed [*I* > 2σ(*I*)] reflections	18194, 4734, 4284
*R*_int_	0.030
(sin θ/λ)_max_ (Å^−1^)	0.618

Refinement	
*R*[*F* ^2^ > 2σ(*F* ^2^)], *wR*(*F* ^2^), *S*	0.032, 0.083, 1.06
No. of reflections	4734
No. of parameters	425
H-atom treatment	All H-atom parameters refined
Δρ_max_, Δρ_min_ (e Å^−3^)	0.22, −0.21

Computer programs: *APEX3* and *SAINT* (Bruker, 2016 ▸), *SHELXT/5* (Sheldrick, 2015*a* ▸), *SHELXL 2018/3* (Sheldrick, 2015*b* ▸), *DIAMOND* (Brandenburg & Putz, 2012 ▸), and *SHELXTL* (Sheldrick, 2008 ▸).

## supplementary crystallographic information

### Crystal data

**Table:**

C_28_H_27_N_3_O_3_S	*F*(000) = 1024
*M**_r_* = 485.58	*D*_x_ = 1.330 Mg m^−^^3^
Monoclinic, *P*2_1_/*n*	Cu *K*α radiation, λ = 1.54178 Å
*a* = 12.0487 (4) Å	Cell parameters from 9855 reflections
*b* = 13.9821 (5) Å	θ = 4.4–72.5°
*c* = 15.0239 (5) Å	µ = 1.47 mm^−^^1^
β = 106.606 (1)°	*T* = 150 K
*V* = 2425.46 (14) Å^3^	Column, colourless
*Z* = 4	0.26 × 0.14 × 0.08 mm

### Data collection

**Table:**

Bruker D8 VENTURE PHOTON 100 CMOS diffractometer	4734 independent reflections
Radiation source: INCOATEC IµS micro–focus source	4284 reflections with *I* > 2σ(*I*)
Mirror monochromator	*R*_int_ = 0.030
Detector resolution: 10.4167 pixels mm^-1^	θ_max_ = 72.4°, θ_min_ = 4.4°
ω scans	*h* = −14→14
Absorption correction: multi-scan (*SADABS*; Krause *et al.*, 2015)	*k* = −15→17
*T*_min_ = 0.79, *T*_max_ = 0.89	*l* = −17→18
18194 measured reflections	

### Refinement

**Table:**

Refinement on *F*^2^	Secondary atom site location: difference Fourier map
Least-squares matrix: full	Hydrogen site location: difference Fourier map
*R*[*F*^2^ > 2σ(*F*^2^)] = 0.032	All H-atom parameters refined
*wR*(*F*^2^) = 0.083	*w* = 1/[σ^2^(*F*_o_^2^) + (0.0386*P*)^2^ + 0.7971*P*] where *P* = (*F*_o_^2^ + 2*F*_c_^2^)/3
*S* = 1.06	(Δ/σ)_max_ = 0.001
4734 reflections	Δρ_max_ = 0.22 e Å^−^^3^
425 parameters	Δρ_min_ = −0.21 e Å^−^^3^
0 restraints	Extinction correction: *SHELXL 2018/3* (Sheldrick, 2015*b*), Fc^*^=kFc[1+0.001xFc^2^λ^3^/sin(2θ)]^-1/4^
Primary atom site location: dual	Extinction coefficient: 0.0039 (2)

### Special details

**Table:**

Geometry. All esds (except the esd in the dihedral angle between two l.s. planes) are estimated using the full covariance matrix. The cell esds are taken into account individually in the estimation of esds in distances, angles and torsion angles; correlations between esds in cell parameters are only used when they are defined by crystal symmetry. An approximate (isotropic) treatment of cell esds is used for estimating esds involving l.s. planes.
Refinement. Refinement of F^2^ against ALL reflections. The weighted R-factor wR and goodness of fit S are based on F^2^, conventional R-factors R are based on F, with F set to zero for negative F^2^. The threshold expression of F^2^ > 2sigma(F^2^) is used only for calculating R-factors(gt) etc. and is not relevant to the choice of reflections for refinement. R-factors based on F^2^ are statistically about twice as large as those based on F, and R- factors based on ALL data will be even larger.

### Fractional atomic coordinates and isotropic or equivalent isotropic displacement parameters (Å^2^)

**Table:**

	*x*	*y*	*z*	*U*_iso_*/*U*_eq_	
S1	0.58184 (3)	0.71687 (2)	0.54963 (2)	0.02702 (10)	
O1	0.55415 (10)	0.41602 (8)	0.04888 (7)	0.0389 (3)	
O2	0.43190 (8)	0.56327 (6)	0.13750 (6)	0.02221 (19)	
H2A	0.4361 (17)	0.5558 (14)	0.0816 (14)	0.053 (5)*	
O3	0.80915 (12)	0.76450 (8)	0.74784 (7)	0.0463 (3)	
N1	0.69752 (9)	0.60562 (7)	0.46015 (7)	0.0226 (2)	
N2	0.29604 (10)	0.63554 (10)	0.43743 (8)	0.0363 (3)	
N3	0.84869 (10)	0.64010 (8)	0.66349 (8)	0.0276 (2)	
H3A	0.8267 (15)	0.6163 (12)	0.6064 (12)	0.036 (4)*	
C1	0.61831 (10)	0.44233 (8)	0.24855 (8)	0.0192 (2)	
H1	0.6482 (13)	0.4819 (11)	0.2084 (10)	0.024 (4)*	
C2	0.50155 (10)	0.40139 (8)	0.18906 (8)	0.0201 (2)	
H2	0.4796 (12)	0.3479 (10)	0.2233 (10)	0.020 (3)*	
C3	0.40183 (10)	0.47505 (8)	0.17204 (8)	0.0195 (2)	
C4	0.38587 (10)	0.49849 (9)	0.26679 (8)	0.0198 (2)	
H4A	0.3566 (13)	0.4409 (11)	0.2914 (10)	0.026 (4)*	
H4B	0.3263 (14)	0.5499 (11)	0.2593 (11)	0.029 (4)*	
C5	0.49669 (10)	0.53100 (8)	0.33547 (8)	0.0184 (2)	
C6	0.49119 (10)	0.59248 (8)	0.40793 (8)	0.0203 (2)	
C7	0.59357 (11)	0.63014 (8)	0.46686 (8)	0.0211 (2)	
C8	0.70418 (10)	0.54452 (8)	0.39243 (8)	0.0217 (2)	
C9	0.60604 (10)	0.50545 (8)	0.32793 (8)	0.0190 (2)	
C10	0.69924 (10)	0.35759 (8)	0.27879 (8)	0.0207 (2)	
C11	0.68696 (11)	0.29760 (9)	0.34899 (9)	0.0260 (3)	
H11	0.6323 (14)	0.3128 (12)	0.3811 (11)	0.031 (4)*	
C12	0.75677 (13)	0.21739 (10)	0.37340 (11)	0.0376 (3)	
H12	0.7497 (16)	0.1783 (14)	0.4238 (13)	0.049 (5)*	
C13	0.83774 (13)	0.19569 (10)	0.32770 (13)	0.0425 (4)	
H13	0.8869 (17)	0.1427 (15)	0.3467 (14)	0.055 (5)*	
C14	0.85014 (13)	0.25445 (11)	0.25757 (12)	0.0401 (4)	
H14	0.9087 (17)	0.2423 (14)	0.2239 (13)	0.048 (5)*	
C15	0.78155 (12)	0.33535 (10)	0.23292 (10)	0.0305 (3)	
H15	0.7883 (16)	0.3783 (13)	0.1832 (13)	0.046 (5)*	
C16	0.51833 (11)	0.36283 (9)	0.09888 (8)	0.0265 (3)	
C17	0.49321 (16)	0.25966 (11)	0.07668 (11)	0.0392 (4)	
H17A	0.5448 (18)	0.2227 (14)	0.1245 (14)	0.052 (5)*	
H17B	0.5020 (18)	0.2458 (15)	0.0139 (15)	0.060 (6)*	
H17C	0.4129 (19)	0.2421 (15)	0.0793 (14)	0.056 (6)*	
C18	0.29017 (12)	0.43558 (10)	0.10695 (9)	0.0268 (3)	
H18A	0.2702 (14)	0.3705 (12)	0.1262 (11)	0.034 (4)*	
H18B	0.2960 (14)	0.4305 (11)	0.0429 (12)	0.032 (4)*	
H18C	0.2254 (14)	0.4804 (12)	0.1047 (11)	0.033 (4)*	
C19	0.38188 (11)	0.61752 (9)	0.42231 (8)	0.0242 (3)	
C20	0.82558 (12)	0.52338 (11)	0.39007 (11)	0.0325 (3)	
H20A	0.8471 (16)	0.4556 (14)	0.4093 (13)	0.048 (5)*	
H20B	0.8298 (17)	0.5294 (14)	0.3262 (14)	0.052 (5)*	
H20C	0.8796 (15)	0.5671 (13)	0.4323 (12)	0.038 (4)*	
C21	0.72565 (13)	0.76893 (9)	0.58406 (9)	0.0289 (3)	
H21A	0.7616 (15)	0.7601 (12)	0.5336 (12)	0.034 (4)*	
H21B	0.7122 (14)	0.8361 (12)	0.5963 (11)	0.034 (4)*	
C22	0.79955 (12)	0.72541 (9)	0.67374 (9)	0.0284 (3)	
C23	0.91089 (11)	0.57882 (10)	0.73536 (9)	0.0262 (3)	
C24	0.92081 (13)	0.48300 (11)	0.71326 (10)	0.0331 (3)	
H24	0.8859 (15)	0.4621 (13)	0.6506 (12)	0.039 (4)*	
C25	0.97905 (15)	0.41944 (12)	0.78136 (12)	0.0416 (4)	
H25	0.9834 (17)	0.3516 (15)	0.7626 (14)	0.055 (5)*	
C26	1.02900 (13)	0.45021 (12)	0.87153 (11)	0.0388 (3)	
H26	1.0692 (16)	0.4050 (13)	0.9198 (13)	0.045 (5)*	
C27	1.02052 (13)	0.54563 (12)	0.89285 (10)	0.0351 (3)	
H27	1.0570 (15)	0.5694 (13)	0.9570 (13)	0.044 (5)*	
C28	0.96158 (12)	0.61022 (11)	0.82593 (9)	0.0304 (3)	
H28	0.9531 (14)	0.6768 (13)	0.8419 (11)	0.037 (4)*	

### Atomic displacement parameters (Å^2^)

**Table:**

	*U*^11^	*U*^22^	*U*^33^	*U*^12^	*U*^13^	*U*^23^
S1	0.03021 (18)	0.02522 (17)	0.02499 (17)	0.00004 (12)	0.00686 (13)	−0.00720 (11)
O1	0.0576 (7)	0.0405 (6)	0.0222 (5)	0.0128 (5)	0.0172 (5)	0.0055 (4)
O2	0.0282 (5)	0.0208 (4)	0.0193 (4)	0.0033 (3)	0.0096 (3)	0.0042 (3)
O3	0.0735 (8)	0.0346 (6)	0.0253 (5)	0.0090 (5)	0.0050 (5)	−0.0072 (4)
N1	0.0235 (5)	0.0207 (5)	0.0229 (5)	−0.0007 (4)	0.0055 (4)	−0.0007 (4)
N2	0.0281 (6)	0.0549 (8)	0.0271 (6)	0.0031 (5)	0.0096 (5)	−0.0098 (5)
N3	0.0293 (6)	0.0301 (6)	0.0211 (5)	−0.0012 (5)	0.0037 (4)	−0.0048 (4)
C1	0.0219 (6)	0.0191 (5)	0.0182 (5)	0.0013 (4)	0.0081 (5)	0.0023 (4)
C2	0.0238 (6)	0.0189 (5)	0.0170 (5)	0.0027 (5)	0.0050 (5)	0.0012 (4)
C3	0.0219 (6)	0.0193 (5)	0.0170 (5)	0.0019 (4)	0.0052 (4)	0.0015 (4)
C4	0.0196 (6)	0.0227 (6)	0.0176 (5)	−0.0008 (5)	0.0061 (5)	0.0006 (4)
C5	0.0218 (6)	0.0169 (5)	0.0169 (5)	0.0003 (4)	0.0062 (4)	0.0036 (4)
C6	0.0223 (6)	0.0206 (5)	0.0186 (5)	0.0018 (5)	0.0068 (4)	0.0017 (4)
C7	0.0267 (6)	0.0185 (5)	0.0182 (5)	0.0007 (5)	0.0063 (5)	0.0019 (4)
C8	0.0221 (6)	0.0198 (5)	0.0232 (6)	−0.0004 (5)	0.0066 (5)	0.0008 (4)
C9	0.0220 (6)	0.0167 (5)	0.0189 (5)	0.0011 (4)	0.0070 (5)	0.0024 (4)
C10	0.0199 (6)	0.0193 (5)	0.0218 (6)	0.0008 (4)	0.0043 (4)	−0.0026 (4)
C11	0.0250 (6)	0.0261 (6)	0.0243 (6)	0.0016 (5)	0.0028 (5)	0.0034 (5)
C12	0.0354 (8)	0.0261 (7)	0.0415 (8)	0.0009 (6)	−0.0049 (6)	0.0092 (6)
C13	0.0294 (7)	0.0235 (7)	0.0632 (10)	0.0078 (6)	−0.0053 (7)	−0.0087 (7)
C14	0.0268 (7)	0.0372 (8)	0.0558 (10)	0.0051 (6)	0.0109 (7)	−0.0174 (7)
C15	0.0273 (7)	0.0316 (7)	0.0355 (7)	0.0020 (5)	0.0134 (6)	−0.0055 (6)
C16	0.0286 (7)	0.0300 (6)	0.0183 (6)	0.0114 (5)	0.0024 (5)	0.0000 (5)
C17	0.0491 (10)	0.0322 (7)	0.0328 (8)	0.0075 (7)	0.0062 (7)	−0.0112 (6)
C18	0.0248 (7)	0.0293 (7)	0.0232 (6)	0.0008 (5)	0.0021 (5)	−0.0040 (5)
C19	0.0270 (7)	0.0285 (6)	0.0171 (5)	−0.0007 (5)	0.0062 (5)	−0.0035 (5)
C20	0.0209 (6)	0.0347 (8)	0.0412 (8)	−0.0030 (6)	0.0075 (6)	−0.0113 (6)
C21	0.0362 (7)	0.0226 (6)	0.0257 (6)	−0.0055 (5)	0.0055 (6)	−0.0021 (5)
C22	0.0337 (7)	0.0254 (6)	0.0245 (6)	−0.0065 (5)	0.0060 (5)	−0.0031 (5)
C23	0.0196 (6)	0.0333 (7)	0.0261 (6)	−0.0030 (5)	0.0071 (5)	−0.0016 (5)
C24	0.0313 (7)	0.0354 (7)	0.0314 (7)	0.0014 (6)	0.0069 (6)	−0.0060 (6)
C25	0.0431 (9)	0.0369 (8)	0.0443 (9)	0.0078 (7)	0.0116 (7)	−0.0006 (7)
C26	0.0348 (8)	0.0468 (9)	0.0349 (8)	0.0091 (7)	0.0101 (6)	0.0084 (7)
C27	0.0294 (7)	0.0492 (9)	0.0262 (7)	−0.0012 (6)	0.0073 (6)	0.0006 (6)
C28	0.0283 (7)	0.0358 (7)	0.0263 (7)	−0.0038 (6)	0.0065 (5)	−0.0032 (5)

### Geometric parameters (Å, º)

**Table:**

S1—C7	1.7712 (12)	C11—H11	0.945 (17)
S1—C21	1.8132 (14)	C12—C13	1.378 (2)
O1—C16	1.2203 (17)	C12—H12	0.957 (19)
O2—C3	1.4245 (14)	C13—C14	1.378 (3)
O2—H2A	0.86 (2)	C13—H13	0.94 (2)
O3—C22	1.2157 (17)	C14—C15	1.387 (2)
N1—C7	1.3299 (16)	C14—H14	0.99 (2)
N1—C8	1.3484 (16)	C15—H15	0.980 (19)
N2—C19	1.1486 (17)	C16—C17	1.492 (2)
N3—C22	1.3599 (18)	C17—H17A	0.96 (2)
N3—C23	1.4138 (17)	C17—H17B	1.00 (2)
N3—H3A	0.887 (18)	C17—H17C	1.01 (2)
C1—C10	1.5192 (16)	C18—H18A	1.004 (17)
C1—C9	1.5244 (16)	C18—H18B	0.987 (17)
C1—C2	1.5450 (16)	C18—H18C	0.995 (17)
C1—H1	0.961 (15)	C20—H20A	1.00 (2)
C2—C16	1.5245 (16)	C20—H20B	0.98 (2)
C2—C3	1.5473 (16)	C20—H20C	0.981 (18)
C2—H2	0.986 (14)	C21—C22	1.5141 (19)
C3—C18	1.5228 (17)	C21—H21A	0.982 (18)
C3—C4	1.5255 (16)	C21—H21B	0.980 (17)
C4—C5	1.5056 (16)	C23—C24	1.394 (2)
C4—H4A	0.992 (16)	C23—C28	1.3938 (18)
C4—H4B	0.999 (16)	C24—C25	1.385 (2)
C5—C9	1.4006 (16)	C24—H24	0.960 (18)
C5—C6	1.4034 (16)	C25—C26	1.384 (2)
C6—C7	1.3995 (17)	C25—H25	1.00 (2)
C6—C19	1.4374 (17)	C26—C27	1.383 (2)
C8—C9	1.4074 (17)	C26—H26	0.979 (19)
C8—C20	1.5026 (18)	C27—C28	1.387 (2)
C10—C11	1.3882 (18)	C27—H27	0.995 (18)
C10—C15	1.3949 (18)	C28—H28	0.974 (18)
C11—C12	1.3869 (19)		
			
C7—S1—C21	102.34 (6)	C12—C13—H13	119.8 (12)
C3—O2—H2A	110.0 (13)	C13—C14—C15	120.26 (14)
C7—N1—C8	118.75 (11)	C13—C14—H14	122.4 (11)
C22—N3—C23	126.73 (11)	C15—C14—H14	117.3 (11)
C22—N3—H3A	114.8 (11)	C14—C15—C10	120.22 (14)
C23—N3—H3A	117.3 (11)	C14—C15—H15	122.0 (11)
C10—C1—C9	114.52 (9)	C10—C15—H15	117.8 (11)
C10—C1—C2	106.53 (9)	O1—C16—C17	122.45 (13)
C9—C1—C2	113.03 (10)	O1—C16—C2	119.45 (12)
C10—C1—H1	108.2 (9)	C17—C16—C2	118.08 (12)
C9—C1—H1	107.2 (9)	C16—C17—H17A	108.0 (12)
C2—C1—H1	107.0 (9)	C16—C17—H17B	109.3 (12)
C16—C2—C1	108.36 (10)	H17A—C17—H17B	112.5 (17)
C16—C2—C3	112.49 (9)	C16—C17—H17C	111.2 (12)
C1—C2—C3	112.62 (9)	H17A—C17—H17C	105.3 (16)
C16—C2—H2	108.6 (8)	H17B—C17—H17C	110.6 (16)
C1—C2—H2	108.4 (8)	C3—C18—H18A	112.6 (9)
C3—C2—H2	106.2 (8)	C3—C18—H18B	110.8 (9)
O2—C3—C18	110.48 (10)	H18A—C18—H18B	107.8 (13)
O2—C3—C4	105.48 (9)	C3—C18—H18C	109.5 (9)
C18—C3—C4	110.69 (10)	H18A—C18—H18C	109.3 (13)
O2—C3—C2	111.73 (9)	H18B—C18—H18C	106.7 (13)
C18—C3—C2	111.51 (10)	N2—C19—C6	177.04 (14)
C4—C3—C2	106.72 (9)	C8—C20—H20A	110.6 (11)
C5—C4—C3	112.38 (10)	C8—C20—H20B	109.3 (11)
C5—C4—H4A	109.2 (9)	H20A—C20—H20B	106.3 (15)
C3—C4—H4A	109.1 (9)	C8—C20—H20C	109.4 (10)
C5—C4—H4B	109.1 (9)	H20A—C20—H20C	109.5 (15)
C3—C4—H4B	109.2 (9)	H20B—C20—H20C	111.7 (15)
H4A—C4—H4B	107.7 (12)	C22—C21—S1	111.32 (9)
C9—C5—C6	118.23 (11)	C22—C21—H21A	111.0 (10)
C9—C5—C4	122.55 (10)	S1—C21—H21A	109.0 (10)
C6—C5—C4	119.16 (10)	C22—C21—H21B	107.8 (10)
C7—C6—C5	119.52 (11)	S1—C21—H21B	104.0 (10)
C7—C6—C19	119.54 (11)	H21A—C21—H21B	113.5 (13)
C5—C6—C19	120.94 (11)	O3—C22—N3	124.57 (13)
N1—C7—C6	122.27 (11)	O3—C22—C21	120.80 (13)
N1—C7—S1	119.70 (9)	N3—C22—C21	114.59 (11)
C6—C7—S1	117.97 (9)	C24—C23—C28	119.47 (13)
N1—C8—C9	123.09 (11)	C24—C23—N3	117.61 (12)
N1—C8—C20	114.24 (11)	C28—C23—N3	122.92 (12)
C9—C8—C20	122.65 (11)	C25—C24—C23	120.04 (14)
C5—C9—C8	118.03 (11)	C25—C24—H24	121.1 (11)
C5—C9—C1	120.98 (10)	C23—C24—H24	118.9 (11)
C8—C9—C1	120.87 (10)	C26—C25—C24	120.68 (15)
C11—C10—C15	119.07 (12)	C26—C25—H25	122.0 (12)
C11—C10—C1	119.99 (11)	C24—C25—H25	117.3 (12)
C15—C10—C1	120.82 (11)	C27—C26—C25	119.10 (14)
C12—C11—C10	120.08 (13)	C27—C26—H26	120.3 (11)
C12—C11—H11	120.3 (10)	C25—C26—H26	120.6 (11)
C10—C11—H11	119.6 (10)	C26—C27—C28	121.13 (14)
C13—C12—C11	120.58 (15)	C26—C27—H27	120.4 (10)
C13—C12—H12	120.3 (11)	C28—C27—H27	118.5 (11)
C11—C12—H12	119.1 (12)	C27—C28—C23	119.57 (14)
C14—C13—C12	119.79 (13)	C27—C28—H28	120.8 (10)
C14—C13—H13	120.3 (12)	C23—C28—H28	119.6 (10)
			
C10—C1—C2—C16	−68.29 (11)	C20—C8—C9—C1	−2.86 (18)
C9—C1—C2—C16	165.08 (9)	C10—C1—C9—C5	−129.80 (11)
C10—C1—C2—C3	166.61 (9)	C2—C1—C9—C5	−7.55 (15)
C9—C1—C2—C3	39.99 (13)	C10—C1—C9—C8	54.15 (15)
C16—C2—C3—O2	−71.57 (13)	C2—C1—C9—C8	176.41 (10)
C1—C2—C3—O2	51.24 (12)	C9—C1—C10—C11	49.93 (15)
C16—C2—C3—C18	52.61 (14)	C2—C1—C10—C11	−75.80 (13)
C1—C2—C3—C18	175.42 (10)	C9—C1—C10—C15	−134.18 (12)
C16—C2—C3—C4	173.61 (10)	C2—C1—C10—C15	100.09 (13)
C1—C2—C3—C4	−63.58 (12)	C15—C10—C11—C12	0.68 (19)
O2—C3—C4—C5	−64.66 (12)	C1—C10—C11—C12	176.64 (12)
C18—C3—C4—C5	175.83 (10)	C10—C11—C12—C13	−0.8 (2)
C2—C3—C4—C5	54.32 (12)	C11—C12—C13—C14	0.5 (2)
C3—C4—C5—C9	−24.80 (15)	C12—C13—C14—C15	0.1 (2)
C3—C4—C5—C6	152.52 (10)	C13—C14—C15—C10	−0.2 (2)
C9—C5—C6—C7	3.36 (16)	C11—C10—C15—C14	−0.16 (19)
C4—C5—C6—C7	−174.07 (10)	C1—C10—C15—C14	−176.09 (12)
C9—C5—C6—C19	−176.81 (11)	C1—C2—C16—O1	−57.14 (15)
C4—C5—C6—C19	5.76 (17)	C3—C2—C16—O1	68.03 (15)
C8—N1—C7—C6	2.00 (17)	C1—C2—C16—C17	121.08 (13)
C8—N1—C7—S1	−175.41 (9)	C3—C2—C16—C17	−113.75 (13)
C5—C6—C7—N1	−3.85 (18)	C7—S1—C21—C22	−98.03 (10)
C19—C6—C7—N1	176.32 (11)	C23—N3—C22—O3	5.2 (2)
C5—C6—C7—S1	173.60 (9)	C23—N3—C22—C21	−172.63 (12)
C19—C6—C7—S1	−6.23 (15)	S1—C21—C22—O3	−96.36 (14)
C21—S1—C7—N1	13.60 (11)	S1—C21—C22—N3	81.57 (14)
C21—S1—C7—C6	−163.92 (9)	C22—N3—C23—C24	158.95 (13)
C7—N1—C8—C9	0.24 (17)	C22—N3—C23—C28	−20.9 (2)
C7—N1—C8—C20	178.81 (11)	C28—C23—C24—C25	1.0 (2)
C6—C5—C9—C8	−1.26 (16)	N3—C23—C24—C25	−178.86 (13)
C4—C5—C9—C8	176.08 (10)	C23—C24—C25—C26	−0.7 (2)
C6—C5—C9—C1	−177.42 (10)	C24—C25—C26—C27	−0.3 (2)
C4—C5—C9—C1	−0.08 (16)	C25—C26—C27—C28	1.0 (2)
N1—C8—C9—C5	−0.58 (17)	C26—C27—C28—C23	−0.6 (2)
C20—C8—C9—C5	−179.02 (12)	C24—C23—C28—C27	−0.4 (2)
N1—C8—C9—C1	175.58 (10)	N3—C23—C28—C27	179.51 (13)

### Hydrogen-bond geometry (Å, º)

*Cg*3 is the centroid of the C10–C15 benzene ring.

**Table:**

*D*—H···*A*	*D*—H	H···*A*	*D*···*A*	*D*—H···*A*
O2—H2*A*···O1^i^	0.86 (2)	2.04 (2)	2.8674 (13)	161.9 (19)
N3—H3*A*···N1	0.887 (18)	2.306 (18)	3.1148 (15)	151.6 (15)
C13—H13···O2^ii^	0.94 (2)	2.40 (2)	3.2520 (19)	150.5 (17)
C20—H20*A*···*Cg*3	1.00 (2)	2.975 (19)	3.6866 (16)	128.7 (13)

Symmetry codes: (i) −*x*+1, −*y*+1, −*z*; (ii) −*x*+3/2, *y*−1/2, −*z*+1/2.
